# Crystallization Behavior and Quality of Frozen Meat

**DOI:** 10.3390/foods10112707

**Published:** 2021-11-05

**Authors:** David S. Dang, Luis J. Bastarrachea, Silvana Martini, Sulaiman K. Matarneh

**Affiliations:** Department of Nutrition, Dietetics and Food Sciences, Utah State University, Logan, UT 84322, USA; david.dang@usu.edu (D.S.D.); luis.bastarrachea@usu.edu (L.J.B.); silvana.martini@usu.edu (S.M.)

**Keywords:** preservation, crystallization, freezing/thawing, meat quality, freezing technologies

## Abstract

Preservation of meat through freezing entails the use of low temperatures to extend a product’s shelf-life, mainly by reducing the rate of microbial spoilage and deterioration reactions. Characteristics of meat that are important to be preserve include tenderness, water holding capacity, color, and flavor. In general, freezing improves meat tenderness, but negatively impacts other quality attributes. The extent to which these attributes are affected depends on the ice crystalline size and distribution, which itself is governed by freezing rate and storage temperature and duration. Although novel technology has made it possible to mitigate the negative effects of freezing, the complex nature of muscle tissue makes it difficult to accurately and consistently predict outcome of meat quality following freezing. This review provides an overview of the current understanding of energy and heat transfer during freezing and its effect on meat quality. Furthermore, the review provides an overview of the current novel technologies utilized to improve the freezing process.

## 1. Introduction

Preserving food to extend its shelf-life has been practiced for several millennia. At its roots, food preservation involves altering the product’s inherent properties, mainly pH and water activity (A_w_), in order to inhibit the growth of pathogenic microorganisms, molds, and spores. Preservation techniques are also used to control chemical reactions involved in deteriorating food products, such as lipolysis, lipid oxidation, and proteolysis [1]. An example where both intrinsic hurdles (pH and A_w_) are modified is during the processing of semidry and dry fermented meat products, in which the product is typically inoculated with a microbial culture to lower the pH, and subsequently dried (lowering A_w_) [2]. However, alteration of the product’s inherent properties to this extent means that it is no longer viewed as “fresh”, a term generally preferred by consumers to be associated with their food [3], particularly concerning meat products. Alternatively, food products can be preserved without the need of making significant changes to their inherent properties when stored at very low temperatures. Although preservation through freezing is applicable to a variety of food commodities, this review focuses on the freezing of fresh meat.

Due to the large amount of water in fresh meat, ~60−70% on a wet basis [4], meat products are prone to microbial spoilage and chemical reactions that can negatively affect aspects of meat quality, such as its color, texture, and flavor [5]. Freezing is extensively utilized by the meat industry as a method of preservation during transport and storage. Nonetheless, freezing can adversely affect the very same quality characteristics that it was meant to preserve, resulting in products that are dissatisfying to consumers [6]. Thus, extensive time and research have been dedicated to understanding the physical and biochemical changes occurring in meat during the freezing process and the etiology of these changes [7]. The scope of this review focuses on the perseveration of fresh meat products through the use of freezing, the effects of freezing on meat quality, and the technological advances that have been made to improve the quality of frozen meat products. However, before we discuss these topics, we must have a reasonable understanding of the process of freezing and crystallization behavior of water.

## 2. Freezing and Crystallization Behavior of Water

By definition, the process of freezing refers to the decrease in molecular motion of existing molecules within an environment [8]. In muscle food, this mainly refers to the decrease in random motion of water molecules that exist within the tissue. The freezing of water in meat can be summarized into three distinct chronological stages: (1) the cooling of the product to its freezing point, (2) the phase transition stage in which latent heat is removed, and (3) the product reaching the final temperature of storage (tempering) [9]. It is during the transition stage that water molecules are oriented in a crystalline manner, and ice crystals are formed.

Within the crystallization stage, the phenomenon of nucleation and crystal growth occurs. In the context of water freezing, nucleation refers to the formation of an “embryo”, or nucleus ice crystal, that can later grow into a larger ice crystal [10]. There are two forms of nucleation that can occur, namely primary and secondary nucleation. Primary nucleation refers to the formation of new crystals within the medium in which no preexisting crystals are present. Once a crystalline structure has been formed, a sudden breakage of the structure can catalyze the formation of new crystals. This phenomenon is referred to as secondary nucleation, and requires less energy to form stable nuclei relative to primary nucleation [10]. Similarly, in a complex matrix with various constituents (e.g., proteins and minerals), similar to that of a food system, these constituents can also lower the energy required to form a nucleus, and assist with secondary nucleation [11]. In both cases, supercooling, which is defined as the subtle decrease in temperature as a result of the initial arrangement of water crystals, is the driving force necessary for nucleation [12]. The rate of nucleation (*β*) for both primary and secondary nucleation can be modeled using Equation (1), where Δ*G_c_* is critical free energy for nucleation, *k* is the Boltzman constant (1.380649 × 10^−23^ J/K), *T* is temperature, *z* is the Zeldovich factor (which is defined by the relationship between the number of nuclei in both equilibrium and steady-state distribution with respect to surface tension temperature, concentration, Avogadro number, and surface area), f* is the frequency of monomer attached to a nucleus, and *C*_0_ is the number of nucleation sites [9]. In summary, Equation (1) implies that the rate of nucleation is a statistical phenomenon, with a higher probability for nucleation to occurring in a system with greater supercooling.

(1) $$\beta =zf^{*}C_{o}exp\;\left(\frac{\Delta G_{c}}{kT}\right)$$

Once formed, the nuclei provide a foundation for which further crystallization can occur, thereby allowing the crystals to grow [13]. Nucleation and crystal growth are two inversely related phenomena that are driven by the rate of supercooling, with more nucleation occurring at greater supercooling rates the smaller the crystal sizes. Therefore, the rate of supercooling is a key factor to consider when determining crystal growth, as it can dictate ice crystal size, distribution, and morphology [9]. Equation (2) is a theoretical model that incorporates supercooling as one of the main determinants of crystalline growth, where *G* is defined as the rate of crystal growth (°C/min), Δ*T_s_* is the supercooling temperature (freezing point temperature [T_f_]—nucleation temperature [T_n_]), and β (growth constant) and *n* (growth order) are constants that are experimentally determined [9]. For a more in-depth explanation of nucleation and growth of water crystals, refer to a review by Kiani and Sun [9], which thoroughly describes modeling approaches for nucleation and crystal growth.

(2) $$G=\beta {\left(\Delta T_{s}\right)}^{n}$$

## 3. Energy Balance and Heat Transfer during Water Crystallization in Meat

In general, the thermal center of a product is a point of reference for determining when the completion of freezing occurs, and thus establishing the optimal freezing time [14]. The alternative to this would be to take a series of temperature measurements across the entire object, and establishing an average mass temperature. However, the average mass temperature has the disadvantage of requiring extensive data collection in order to provide precise and accurate estimations [14,15]. Therefore, thermal center measurements are used more often when establishing freezing time [16]. The total time required for a product to reach the set freezing temperature at the thermal center is known as the effective/standard freezing time [17]. Meat products vary in size and shape, and subsequently the rate of freezing also varies considerably between products. Thus, an effort has been made to establish better freezing practices in order to achieve high-quality products. In this regard, modeling approaches have been created to predict the freezing time for various products. Primarily, heat and mass transfer modeling have been used [18,19,20].

### 3.1. Energy Balance

Equation (3) can be used to calculate the amount of heat to be removed from any food sample:

(3) $${Q=m}_{s}c_{p,s}\left(T_{c}{-\;T}_{i}\right){+m}_{w}c_{p,w}\left(T_{c}{-\;T}_{i}\right){\;-\;m}_{w}\lambda_{w}{+m}_{s}c_{p,s}\left(T_{f}{-\;T}_{c}\right){+m}_{w}c_{p,w}\left(T_{f}{-\;T}_{c}\right)$$

where *Q* is the heat to be removed (kJ), *m_s_* is the mass of solids in the sample (kg), *c_p,s_* and *c_p,w_* are the values of specific heat of the solids and water (kJ/(kg·°C)), respectively, *λ_w_* is the enthalpy of crystallization or fusion of water at the crystallization temperature (kJ/kg), *T_i_* is the initial temperature of the sample above the crystallization temperature (°C), *T_c_* is the water crystallization temperature (°C), and *T_f_* is the final temperature of the sample below the water crystallization temperature (°C). It is important to emphasize that the values of specific heat may not be constant in a given range of temperatures, and the specific heat of a substance can change with a change in phase [21,22].

As can be expected, the presence of solutes in food samples can substantially alter the crystallization temperature of the water they contain, causing it to have a lower value than the crystallization temperature of pure water. In general, the deviation of the crystallization temperature from that of pure water depends on the type of solutes present (their molecular weight) and their concentration [23]. Likewise, the values of specific heat of the solids depend on their specific components and their physical state. Several equations have been reported to predict crystallization temperatures of water in foods, and for calculating specific heat values below and above the corresponding crystallization temperature [24,25].

**Figure 1:**
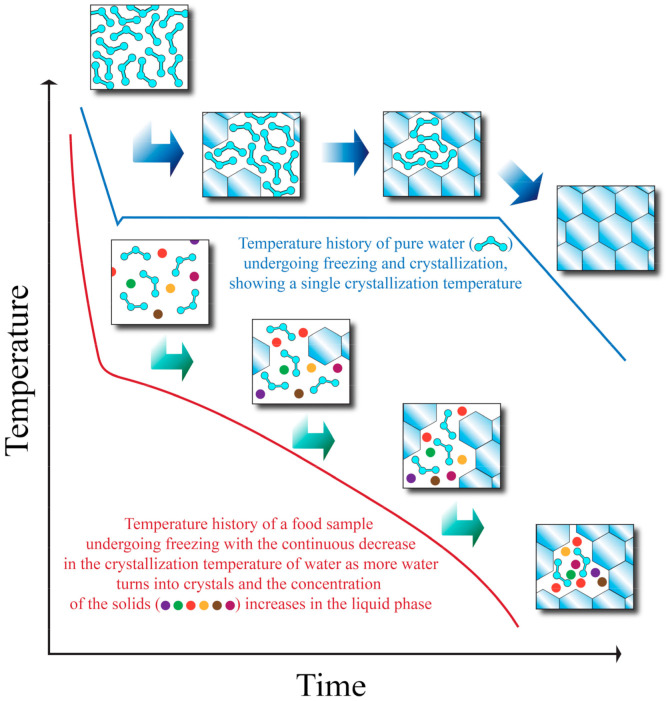
Temperature history of pure water and a food sample during freezing. Adapted from Heldman and Singh [21].

Even though it is possible to predict the thermal properties of foods (such as water crystallization temperature and specific heat) with empirical equations as explained earlier, the realistic amount of total removable heat required to freeze a sample may deviate from the calculated *Q* value of Equation (3) (which can be seen as a theoretical value or a simplification). Figure 1 shows the freezing curves of pure water and a food sample. As heat is removed from the food sample, water starts to crystallize at an initial crystallization temperature. At a certain point, the crystallization temperature begins to decrease due to having a more concentrated solution, and crystallization will come to a stop unless more heat is removed from the system. If more heat is removed, one or more eutectic temperatures (the temperature at which there is a coexistence of liquid and solid phases) may be reached. After the eutectic temperatures have been reached, all of the components exist in solid form, and the temperature may continue to decrease as more heat is removed after all the components have undergone crystallization [21].

### 3.2. Heat Transfer and Water Crystallization in Meat

The rate of heat transfer or freezing rate is influenced by the temperature gradient between the product and the freezing medium, the modes of heat transfer involved (convection, conduction, radiation, or their combination), the shape and size of the product as well as its packaging material, and the thermal properties of the product. The fact that many thermal properties (e.g., specific heat, thermal conductivity, and enthalpies of phase change) vary with changes in temperature can make the determination of rates of heat transfer challenging. This, in turn, has created the need to develop simplified solutions to calculate rates of heat transfer and times required to freeze a product. These solutions usually assume the thermal properties to be constant as a function of temperature [21].

The governing equations of heat transfer due to conduction for regular geometries are shown below. Equation (4) corresponds to Cartesian coordinates (rectangular geometries), Equation (5) corresponds to cylindrical coordinates, and Equation (6) applies to a spherical system [26].

(4) $$k\;\left[\frac{\partial^{2}T}{\partial x^{2}}+\frac{\partial^{2}T}{\partial y^{2\;}}+\frac{\partial^{2}T}{\partial z^{2\;}}\;\right]+\dot{g\;}{=\rho c}_{p}\frac{\partial T}{\partial t}$$

(5) $$k\left[\;\frac{1}{r}\frac{\partial }{\partial r}\left(r\frac{\partial T}{\partial r}\right)+\frac{1}{r^{2}}\left(\;\frac{\partial^{2}T}{\partial \phi^{2}}\;\right)+\frac{\partial^{2}T}{\partial z^{2}}\;\right]{+\dot{g}=\rho c}_{p}\frac{\partial T}{\partial t}$$

(6) $$k\;\left[\;\frac{1}{r^{2}}\frac{\partial }{\partial r}\left(r^{2}\frac{\partial T}{\partial r}\right)+\frac{1}{r^{2}\sin^{2}\theta }\left(\;\frac{\partial^{2}T}{\partial \phi^{2}}\;\right)+\frac{1}{r^{2}\sin\theta }\frac{\partial }{\partial \theta }\left(\sin\theta \frac{\partial T}{\partial \theta }\right)\;\right]+\dot{g\;}{=\rho c}_{p}\frac{\partial T}{\partial t}$$

Equations (4)–(6) relate the rate of heat transfer in the three dimensions of the corresponding coordinate systems and the heat generated within $\dot{g}$ (W/m^3^), with the change in heat content in the corresponding working volume (right side of the equations). If a change in phase (such as water crystallization during freezing) takes place, that would need to be incorporated in the right side of the equations as the total change in enthalpy. To overcome the lack of accuracy from the use of empirical models to predict thermal properties and calculate indirect changes in heat content, thermal analysis seems to be the best approach to determine the exact amount of heat to be removed to reach a defined level of water crystallization. Some of the most frequently used thermal analysis techniques include Differential Scanning Calorimetry (DSC), Differential Thermal Analysis (DTA), and Thermogravimetric Analysis (TG) [27]. Once the total change in enthalpy from a freezing process is determined through any of these thermal analysis techniques, it can be simply taken into account for freezing time calculations. Thermal analyses allow an accurate determination of energy supply or removal requirements [27,28]. Hobani and Elansari [29] examined the enthalpy change from –40 to 40 °C of meat through Modulated Differential Scanning Calorimetry (MDSC). These authors were able to determine the heat content (which accounted for the freezing and crystallization of water) as a function of moisture, and accurately predicted the changes in specific heat along the temperature range studied.

By analytically solving the governing equations above, it is possible to determine the rates of heat transfer and temperature distributions in an object, and if it is established that heat transfer only takes place in one dimension (which in many situations can be a safe assumption), those analytical solutions can be more easily determined. The analytical solutions of the governing equations involve performing the corresponding energy balance in the working volume, applying the known boundary conditions, and solving the differential equation to determine the corresponding integration constants. The analytical solutions are also known as exact solutions since they agree with the boundary conditions of the differential equations [30]. An example of a widely used analytical solution for regular geometries is Plank’s Equation (Equation (7)), which can be used to calculate freezing times assuming that heat transfer is unidimensional [21,23]:

(7) $$t_{F}=\frac{{\rho \lambda }_{w}}{T_{F}-T_{\infty }}\left[\frac{Pa}{h}+\frac{{Ra}^{2}}{k}\right]$$

where *t_F_* is the freezing time, *ρ* is the object’s density, *T_F_* is the initial freezing point, *T*_∞_ is the cooling medium temperature, *a* is the characteristic length of the object (the thickness for the case of a slab, or the diameter in the case of a cylinder or a sphere), *P* and *R* are constants that depend on the object’s geometry (slab, cylinder or sphere), *h* is the convection heat transfer coefficient of the cooling medium (which may need to be determined experimentally), and *k* is the object’s thermal conductivity.

Other analytical, semi-analytical, and empirical methods for freezing time calculation include a modification of Plank’s equation by Cleland and Earle, the method of Lacroix and Castaigne, the method of Pham, the method of Salvadori and Mascheroni, the method of Hung and Thompson, the method of Ilicali and Saglam, the Neumann method, the Tao solutions, the Tien solutions, and the Mott procedure [21,31]. These methods can be applied to objects with regular shapes (slabs, cylinders, or spheres). Other methods have been developed that can be applied to predict freezing times of objects with irregular shapes, and include the methods of Cleland and Earle, Cleland et al., Hossain et al., and Lin et al. [31].

**Figure 2:**
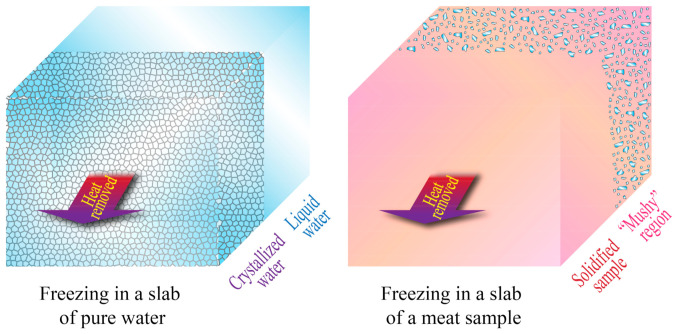
Unidimensional heat transfer and freezing in slabs of pure water and a meat sample. Adapted from Datta [26].

The main advantage of most analytical, semi-analytical, and empirical solutions is their simplicity, but they also have limited applicability in real situations. Many of them require the system or object being studied to have a regular geometry, which may be very unlikely for biological systems. Additionally, many analytical solutions (such as Plank’s equation) assume the physical and thermal properties (such as density, crystallization temperature, thermal conductivity, specific heat, and enthalpies of crystallization) to be constant, which may be an oversimplification that can cause substantial deviations from real measurements, and may not take into consideration the changes in enthalpy due to sensible heat above and below the crystallization temperature. Moreover, analytical solutions assume steady-state heat transfer, a condition that implies that the temperature distribution throughout the system remains constant over time, which is another ideal scenario rarely found in real situations [21,30]. Additionally, for the specific case of meat and other biomaterials (cellular tissues), the complexity of their structure generates an even greater deviation from the results obtained in analytical solutions, which also assume samples or objects with a homogeneous mass. This feature could only be expected in a pure substance. In reality, meat samples may exhibit micro-regions or “mushy” regions where crystallization and/or solidification may take place in separated areas, rather than uniformly [26], as shown in Figure 2.

To overcome the limited accuracy and applicability of analytical solutions, numerical methods can be employed, which involve the division of the studied object or medium into small subdivisions that result in the same number of algebraic equations for the unknown temperatures at the nodes located in the interfaces of such subdivisions. These equations can then be solved through computational methods to determine the temperature distributions within the medium. Some frequently employed numerical methods are the finite difference method, the finite element method, the boundary element method, the finite volume method, and the energy balance method [30,32]. These methods can be applied under steady or unsteady state conditions, thereby allowing for the computation of freezing times. The majority of the currently available computational tools and software use the finite element method and the finite volume method [32,33]. Application of numerical methods also requires the knowledge of the initial and boundary conditions of the process or phenomenon being studied. Some commonly used initial and boundary conditions include the initial or specified temperature boundary condition, the heat flux boundary condition, the convection boundary condition, the radiation boundary condition, the combined radiation and convection boundary condition, the combined heat flux, radiation and convection boundary condition, and the interface boundary condition [30].

The numerical solutions of the partial differential equations (such as Equations (4)–(6)) that describe physical phenomena can be used to perform virtual or computational simulations of a variety of processing operations, including freezing. Knowing the necessary energy to be removed from a food sample to reach a specific temperature (and/or the physical and thermal properties of the studied food item), as well as the environmental conditions (such as convective heat transfer coefficients and freezing medium temperature), it is possible to predict the time-temperature histories. These predicted values can then be validated in a real experiment, which represents substantial savings in time, material, and labor resources [32].

**Figure 3:**
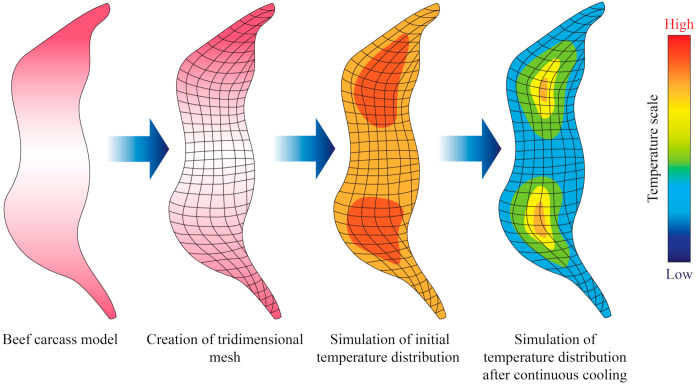
Illustration showing an example of the output obtained from the simulation of cooling or freezing through the solution of the heat transfer differential equations through numerical methods. Adapted from Trujillo and Pham [36].

Sun and Zhu [34] conducted computational simulations to determine the freezing time of beef samples with different muscle fiber orientations (parallel to the heat transfer direction and perpendicular). In their study, heat transfer was assumed to be unidirectional in Cartesian coordinates (Equation (4)), and applied the finite difference method with a Crank–Nicholson formulation. They also took into consideration changes of thermal conductivity with temperature. Overall, their results showed a good fit with the predicted simulation that was utilized. However, other studies took into consideration mass transfer phenomena in their modelling approach, as water may either condensate or evaporate from the surface of meat samples during freezing or thawing. Delgado and Sun [35] applied the explicit finite difference method to perform simulations of simultaneous heat and mass transfer during thawing of mild cured ham samples, and the experimental data exhibited a good fit with the partial differential equation applied. More recently, Trujillo and Pham [36] conducted an evaluation of the chilling process of a beef carcass through Computational Fluid Dynamics (CFD), which involved simultaneous heat and mass transfer. The tridimensional geometry of the beef carcass was built in the software the authors employed through established correlations between the different parts of the carcass (Figure 3). The authors found that the agreement between the predicted and measured data (such as temperature and superficial moisture loss) depended on the part of the carcass, possibly due to local insulating or mass transfer resistance effects.

## 4. Freezing as a Form of Meat Preservation

Meat provides an excellent medium for yeast, molds, and pathogenic bacteria to grow [37], making meat products highly perishable. Typical genera of microorganisms that are frequently found on meat products are: *Acinetobacter*, *Aeromonas*, *Alternaria*, *Enterococcus*, *Micrococcus*, *Manoscus*, *Pseudomonas*, and *Penicillium* [5]. Due to their threat to human health, strict food safety guidelines are followed by the meat industry to control pathogenic microorganisms such as Salmonella and *Escherichia coli* [38]. However, it is important to point out that contamination of meat products does not truly occur until the animal has been harvested and the carcass has been dressed. In fact, the tissue under the animal’s hide is sterile until it is punctured during the harvest process [39]. Further contamination may also take place during carcass fabrication, processing, and handling. In general, microbial contamination occurs on the surface of whole meat cuts, whereas in ground meat, microbial contaminants are distributed throughout the product due to the mixing that occurs during its manufacture.

At the retail and consumer level, meat products can temporarily be stored under refrigeration temperatures for a short period of time before spoilage occurs. On the other hand, frozen meat has an extended shelf-life. While freezing does not eliminate microbial organisms, it does, however, prevent microbial proliferation, thereby reducing the rate of spoilage. However, caution must be taken when thawing a meat product, as it would be prone to microbial growth [7]. During thawing, meat exudes internal moisture to the surface, carrying along with it nutrients for microbial growth [7,40]. This may explain why bacteria on the surface of frozen/thawed meat have a shorter lag phase than those on fresh meat [41,42], implying that the shelf-life of meat products may be reduced once thawed. Therefore, it is recommended by the USDA that frozen meat should be thawed under refrigeration temperatures in order to decrease the risk of spoilage, and to better preserve quality [43]. Although freezing is mainly utilized to limit spoilage of meat, other characteristics of the meat can be influenced by this process.

## 5. Aspects of Meat Quality in Relation to Freezing

**Figure 4:**
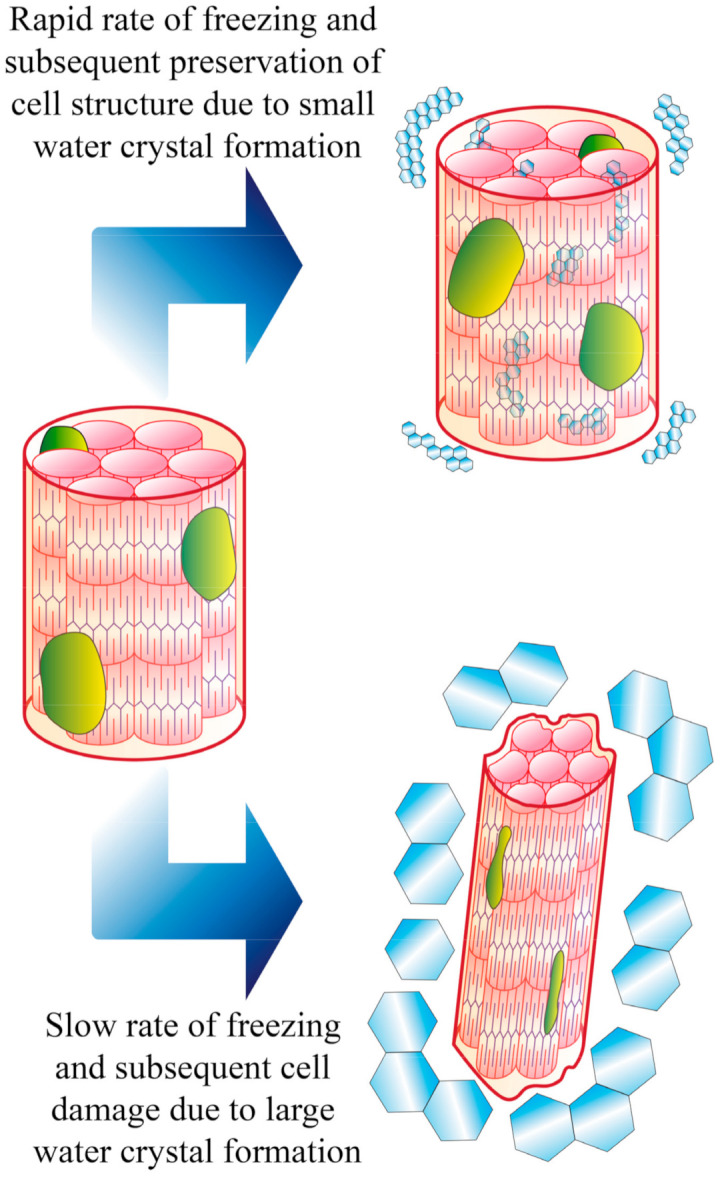
Effects of cell integrity at different speeds of cooling and water crystallization. Adapted from Schudel, et al. [46].

The cooling rate during freezing can greatly influence the microstructure and overall quality of meat (Figure 4). A slow cooling rate promotes the formation of large ice crystals, and consequently a more pronounced deformation to the original cell structure. In meat, large crystals can also mechanically damage myofibrillar structures and cause a decrease in water holding capacity (WHC) [44], which, in turn, negatively impact product’s color, flavor, and juiciness [45]. In contrast, greater cooling rates results in the formation of smaller crystals, and therefore less damage to the cellular structure. For this reason, fast cooling rates are preferred for freezing food products in general, and meat products in particular. Table 1 lists studies that have investigated effects of freezing rate and thawing on the quality of meat, including tenderness, WHC, and color. The effect of freezing/thawing on each of these attributes will be further discussed in the following sections.

In addition to cooling rate, the time of initial exposure to freezing temperatures is another important factor that has to be taken into consideration when freezing meat products. A classic example that demonstrates this aspect is the case of meat that has undergone thaw rigor or thaw contracture. Thaw rigor occurs when a muscle is frozen prior to the completion of rigor mortis and then thawed. The formation of ice crystals upon freezing disrupts the sarcoplasmic reticulum within muscle cells, resulting in a sudden release of calcium into the cytosol after thawing. In the presence of ATP, as in the case of pre-rigor muscle, calcium initiates a strong muscle contraction, leading to a shorter sarcomere length and a tougher product [59]. Hence, it is imperative to freeze meat following the completion of rigor mortis (i.e., ATP depletion), an event that takes 2−4 h in chicken and turkey, 8−12 h in pork, and ~24 h in lamb and beef [60].

Under freezing conditions, meat can be stored for an extended period of time before quality defects are noticeable. Several studies have evaluated the effect of freezing duration on meat quality [53,61,62], showing a generally negative relationship between the two. Vieira, Diaz, Martínez, and García-Cachán [53] found greater water loss and lower L* (lightness), a* (redness), and b* (yellowness) values in beef steaks that were frozen for 90 days than in steaks that were frozen for 30 days. In a different study, Muela, et al. [63] observed a decrease in a* and tenderness in lamb chops that were frozen for 21 months, compared to those frozen for only 1 month. Previous studies have attempted to determine optimal storage duration during freezing [51,55,64]. Soyer, Özalp, Dalmış, and Bilgin [64] reported that chicken stored at −18 °C is stable for up to 3 months before deterioration occurs, whereas it ranges between ~3–21 months for lamb [63,65] and ~42 days–12 months for beef stored at the same temperature [53,61]. Storing meat long-term at a temperature range of −18 °C to −20 °C allows unfrozen water to participate in chemical reactions with other constituents (e.g., proteins and lipids), which leads to a loss of quality [7]. Estévez, et al. [66] recommend that meat should be frozen at −40 °C, as only a small fraction of water is unfrozen at this temperature. Taken together, recommending an ideal storage duration to achieve minimal quality loss is a challenge since factors such as species, freezing rate, and storage temperature also play a role in determining quality [7]. To accurately interpret the effects of freezing on meat quality, Table 1 also contains information on the species and storage temperature and duration that was utilized in each study.

### 5.1. Tenderness

Tenderness is an important quality attribute that determines the overall acceptance of meat products by consumers [67,68]. While some studies reported no affect [52,56] or a negative effect [54,55] of freezing on meat tenderness, the majority of the studies in the literature indicate a positive relationship between freezing and tenderness [50,51,53,69,70]. Lagerstedt, et al. [50] observed ~10 Newton reduction in the Warner–Bratzler Shear Force (WBSF) of beef steaks that were frozen before aging, compared to steaks that were aged but never frozen. Likewise, sensory panelists perceived frozen/thawed beef steaks to be more tender than their fresh counterparts [53]. The positive effect of freezing on tenderness has also been observed in pork [71], chicken [72], and lamb [54]. This enhancement in tenderness has been attributed, in part, to mechanical damage of the myofibrillar structure of meat by the ice crystals [7]. Using transmission electron microscopy, Qi, et al. [73] observed weakened sarcomere M- and Z-lines after 5 freeze-thaw cycles. More importantly, they associated these ultrastructural changes with a decrease in hardness, chewiness, and cohesiveness, which are all textural conditions that are associated with a more tender product. The same effect has also been observed in cooked meat, in which greater destruction to the myofibrillar structure and enhanced tenderness were observed when lamb was cooked and subsequently frozen [74]. In addition to physically disruption, greater tenderness can also be achieved through the degradation of myofibrillar proteins by endogenous proteases (proteolysis) during meat storage. Postmortem proteolysis has been widely considered as one of the most significant events that dictates end-product tenderness [75]. Indeed, Aroeira, et al. [76] and Grayson, et al. [77] showed that enhanced tenderness of frozen/thawed beef steaks was associated with greater postmortem proteolysis.

There are several proteolytic enzyme systems that are involved in postmortem proteolysis, including calcium-dependent proteinases (calpains), cathepsins, and caspases. Among these systems, calpains, more specifically, calpain-1, have been recognized for their role in postmortem tenderization [78]. Calpain-1 is the main protease responsible for the degradation of myofibrillar proteins such as titin, nebulin, troponin-T, and desmin during meat aging, thereby enhancing tenderness [79,80]. Therefore, increased proteolysis in frozen/thawed meat has been postulated to be due to improved calpain-1 activity, presumably through elevating cytosolic calcium concentration as a result of disrupting the sarcoplasmic reticulum by ice crystals. Indeed, Zhang and Ertbjerg [81] reported calcium concentrations of 400–900 µM in a pork *longissimus* muscle that was subjected to freezing and then thawed, a concentration well above the threshold required for activating calpain-1 (3−50 µM calcium for half maximum activity) [82]. In support of this construct, Koohmaraie [83] demonstrated a rapid decrease in the activity of calpastatin (the endogenous inhibitor of calpain-1) isolated from frozen beef *longissimus* muscle, an effect that was also observed in frozen lamb chops [84,85]. The decrease in calpastatin activity following freezing/thawing is likely a function of increased cytosolic calcium concentration. At low calcium concentrations, calpastatin binds to and inhibits calpain-1, whereas at higher calcium concentrations, calpastatin is degraded by calpain-1 and other endogenous proteases, which lowers its inhibitory effect on calpain-1 [86]. To the best of our knowledge, however, no previous research has shown an increase in calpain-1 activity following freezing/thawing, suggesting that more research should be conducted in this area.

While calpain-1 is regarded as the main contributor to meat tenderization during aging, recent reports have suggested that cathepsins and caspases can also contribute to postmortem tenderization [87]. The formation of ice crystals has been shown to disrupt the lysosome (the organelle that houses cathepsins), allowing cathepsins to be released into the sarcoplasm to initiate proteolysis [88]. In support of this, Lee et al. [89] observed higher cathepsin B activity in beef semitendinosus muscle that was frozen for 24 h at −20 °C prior to aging, in comparison to non-frozen samples. To our knowledge, no previous research has investigated the participation of caspases in proteolysis of frozen/thawed meat. However, it has been documented that freezing skeletal muscle tissue disrupts the mitochondria [90], which allows the release of the pro-apoptotic protein cytochrome c into the cytosol. In the cytosol, cytochrome c initiates a cascade of events, ultimately leading to the activation of the intrinsic apoptotic pathway, a process that is necessary for the activation of the caspase system [91,92]. Studies have shown that early disruption of the mitochondria in meat led to greater activity of caspase-3, the main effector caspase involved in apoptosis [93,94]. However, the area of research regarding meat freezing and activation of the caspase system requires additional investigation. Taken together, freezing can induce macro- and micro-level changes in meat, which, in most cases, improves meat tenderness. However, the mechanical damage caused by the ice crystals in the meat can impair its ability to hold onto water [7]. Thus, other meat quality attributes, such as its WHC, have been shown to be adversely affected by the process of freezing [95].

### 5.2. Water Holding Capacity

There are three forms of water in skeletal muscle tissue: bound, immobilized, and free. Bound water represents ~5% of total muscle water, and is tightly bound to muscle proteins, which prevents it from moving to other compartments. This type of water is resistant to freezing and is not easily evaporated during heating [96]. On the other hand, ~85% of myowater is sterically immobilized within the muscle cell, while the remaining 10% are freely moving water held in the extracellular spaces by capillary forces [44]. Both immobilized and free water are susceptible to temperature and pressure changes, and can be lost as purge and drip [96].

Under normal circumstances, the loss of water from postmortem muscle is an inevitable event. This is mainly due to the decline in muscle pH closer to the isoelectric point of muscle proteins (pH 5.1−5.2), and the shrinkage of the myofibrillar lattice driven by rigor mortis [7]. Postharvest handling (i.e., cold storage/freezing) of meat can also further decrease its ability to retain water. Añón and Calvelo [97] reported that, under slow freezing rates, more water is removed from the intracellular space of muscle cells in order to form large ice crystals in the extracellular space, which, in turn, permits for more thaw loss. In addition, large ice crystals are capable of distorting and disrupting muscle cells, facilitating more water to be exuded. Zhang and Ertbjerg [81] observed an increase in purge and cooking loss in pork loins that were frozen at −20 °C and then thawed. Similar findings were also observed in studies that evaluated WHC in frozen/thawed beef [50], lamb [54], and chicken [58]. Interestingly, the rate at which product temperature drops from −1 to −7 °C has been shown to influence the amount of water loss during thawing, with greater freezing rates during this range leads to less water loss [97].

Juiciness is one of the major eating quality attributes that is significantly affected by meat’s WHC. Meat juiciness is generally improved with increasing WHC, and thus the decrease in WHC due to freezing detrimentally affects meat juiciness. Lagerstedt, Enfält, Johansson, and Lundström [50] indicated that sensory panelists perceived cooked beef that was previously frozen to be significantly less juicy than non-frozen cooked steaks. Impaired WHC due to freezing can also negatively impact the functional properties of meat. During sausage manufacturing, WHC dictates final product quality, with greater WHC equating to a juicier and firmer sausages [98]. Furthermore, greater WHC allows for the formation of a more stable meat batter, ensuring uniform textural and visual properties in the final product [99]. Carballo et al. [100] reported increased water loss from a meat batter that was prepared from frozen/thawed pork than those from non-frozen pork, suggesting instable batter. Although the use of partially frozen meat is necessary for controlling temperature and facilitating the cutting and mincing process during sausage manufacturing, more attention should be paid when frozen meats are used due to their impaired WHC [101].

### 5.3. Color

Surface color is one of the most important visual aspects of meat, and can strongly influence consumer’s purchasing decisions [102]. This is largely because consumers associate the color of the meat with its wholesomeness, freshness, and eating quality [103]. Observed meat color is primarily related to the amount and redox state of the heme pigment myoglobin in the meat.

Myoglobin is a protein composed of a single polypeptide chain (globin) bound to a heme prosthetic group. The heme portion of the molecule is embedded in the hydrophobic pocket within the globin, and contains a single iron atom at its center. The iron is coordinated to four pyrrole nitrogen atoms and the imidazole nitrogen of a histidine residue of the globin. Lastly, a sixth coordination site is available for ligand binding and redox reactions. Depending on the nature of the ligand and redox state of the iron, myoglobin can exist in three different forms: deoxymyoglobin, oxymyoglobin, and metmyoglobin. Deoxymyoglobin contains iron in the reduced ferrous form (Fe^2+^), with no ligand bound to its sixth coordination site, and its color appears dark-purplish red. Once diatomic oxygen occupies the sixth binding site of Fe^2+^, the desirable bright cherry-red color of fresh meat, oxymyoglobin, is formed. In comparison, discoloration (browning) of fresh meat results from the accumulation of metmyoglobin, a process that involves the oxidation of ferrous iron to its ferric state (Fe^3+^).

The ability of meat to hold water (i.e., WHC) is fundamental in determining end-product color. As a water-soluble protein, myoglobin is lost along with the fluids that exudate from the meat during the postmortem period, making the product appear lighter. As such, meat that has greater WHC can retain more water, and appears darker and redder in color [103,104]. Several studies have reported that freezing causes the meat to become lighter and less red in color [53,54,56]. Kim, Kim, Seo, Setyabrata, and Kim [47] reported an increase in L* and a decrease in a* values in a thawed pork *longissimus* muscle that was frozen at −20 °C. Aroeira et al. [105] reported lower a* values of beef steaks that were frozen/thawed and then aged for 14 days compared to samples that were not subjected to freezing but aged for the same amount of time. In addition, several authors have reported that freezing alters myoglobin forms in meat [106,107,108]. Aroeira et al. [76] reported 8–22% greater metmyoglobin levels in beef steaks that were frozen at −20 °C and subsequently aged for 14 days than steaks that were not subjected to freezing. In another study where the color of frozen/thawed beef from Nellore and Angus cattle was compared, greater metmyoglobin content in beef from the Nellore cattle was observed [105]. This was attributed to greater polyunsaturated fatty acids in beef from Nellore cattle, which promotes myoglobin oxidation. In a different study, Jeong et al. [107] reported that beef steaks aged after freezing had lower oxymyoglobin content than non-frozen steaks after the same aging period. However, Alvarenga et al. [109] showed greater oxymyoglobin level in lamb steaks that were frozen after aging in comparison to non-frozen samples. This greater oxymyoglobin in frozen/thawed steaks was attributed to the decrease in mitochondrial functionality caused by disrupting ice crystals, thereby increasing the oxygen available for myoglobin.

### 5.4. Flavor

Frozen storage is utilized to minimize the amount of biochemical reactions that can occur in a meat product. However, at suboptimal freezing temperatures, ranging between 0 and −20 °C, a proportion of the myowater remains unfrozen, allowing chemical reactions to occur over time [110]. As more ice crystals are formed, constituents (proteins, carbohydrates, lipids, vitamins, and minerals) within the meat are concentrated in the unfrozen water phase, which increases the probability of lipid and protein oxidation [7]. In addition, disruption of cellular structures by large ice crystals releases oxidizing agents, such as heme and non-heme iron and membrane lipids that accelerate oxidative reactions [64,111]. In this regard, various studies have shown an increase in oxidation during freezing/thawing of meat, and subsequently the development of undesirable flavor, color, and textural changes [64,112,113,114,115]. In particular, poultry meat is highly susceptible to oxidation than meats from other species, due to its high content of unsaturated fatty acids [116]. Moreover, Soyer et al. [64] observed greater protein oxidation in chicken legs (darker in color) compared to the breasts (lighter in color) after 6 months of storage, which was attributed to greater levels of iron and lipid in the legs [117]. However, when Estévez et al. [66] compared protein oxidation between porcine *longissimus* (lighter in color) and psoas major (darker in color) muscles that were frozen at −18 °C and stored for 12 weeks, they found more protein oxidation in the *longissimus*. In this case, increased protein oxidation in the *longissimus* was suggested to be due to lower WHC compared to the *psoas major*.

The number of freeze/thaw cycles is another important factor that influences the extent of protein and lipid oxidation [118,119,120]. Rahman et al. [121] observed a continues increase in lipid oxidation products in beef muscles that underwent three cycles of freezing/thawing. A plausible explanation for this could be that increasing the number of freeze/thaw cycles induces more disruption of cellular components. This, in turn, increases the likelihood of more oxidizing agents to be released and participate in lipid and protein oxidation reactions. In support of this notion, Benjakul and Bauer [122] observed an increase in non-heme iron in fish muscle after every subsequent freeze/thaw cycle. To combat against the loss in quality due to oxidation, storing meat at a temperature of −40 °C or lower has been suggested to limit the occurrence of oxidative reactions [123]. However, for general consumers, it is more common to have commercial freezers that only operate at temperatures close to −20 °C. In this case, minimizing the number of freeze/thaw cycles is recommended.

One of the main concerns regarding lipid and protein oxidation is the development of rancid flavor from the volatile compounds that are generated by oxidation reactions. However, several sensory-based studies indicated that frozen meat does not produce a detectable amount of “off-flavor” for consumers to perceive [55,65,69]. Muela et al. [55] conducted a sensory analysis to compare lamb samples that were frozen using three different freezing methods (air blast [−30 °C], freezing tunnel [−40 °C], and liquid nitrogen [−75 °C]) to non-frozen meat, and observed no differences in sensory perception between frozen and fresh meat. In another study by Hergenreder et al. [69], three different beef muscles (*longissimus thoracis*, *longissimus lumborum*, and *gluteus medius*) that were previously frozen at −28 °C were compared to non-frozen counterparts using sensory analysis. In this study, the sensory panelists did not detect any difference in off-flavor between the frozen and fresh *longissimus thoracis* and *longissimus lumborum* samples, but they were able to detect a slightly higher off-flavor in the frozen *gluteus medius* compared to the non-frozen samples. In general, a threshold concentration of oxidation products is needed before an off-flavor could be detected by a sensory panelist (threshold limit) [124], which probably was not achieved in these two studies.

## 6. Methods for Mitigating the Effects of Freezing and Thawing of Meat Products

Over recent years, novel methodologies of freezing and thawing of meat products have been investigated to achieve better overall meat quality. High-pressure freezing is a method that creates a high supercooling effect that allows for the formation of homogenous ice crystals within the product. At high pressure, ~200−400 MPa, water remains in a liquid state at a temperature below 0 °C, and rapid ice formation occurs upon an immediate drop in pressure [125]. Moreover, type IV ice crystals are formed during high-pressure freezing [126]. These crystals are smaller and denser than water, which reduces their ability to swell (increase in volume) during freezing, thereby reducing the mechanical damage imposed on the surrounding tissues. In support of this, Jia et al. [127] observed ~35% reduction in drip loss of pork *longissimus* samples that were pressurized at 400 MPa and subsequently frozen at −20 °C. Other studies similarly found improvements in the quality of beef [128], chicken [129], and fish [130] samples that were subjected to high-pressure freezing compared to conventional freezing. On the other hand, type I ice crystals are formed when water is frozen at atmospheric pressure. In comparison to type IV ice crystals, type I crystals possess 9-13% greater volume at freezing temperatures ranging from 0 to −20 °C [131]. Thus, high-pressure freezing is a method that can be utilized to mitigate unwanted detriments caused by freezing, specifically from type I ice crystals.

Another novel and potential freezing technology that has been explored over the past decade is static electric field (SEF) freezing. Relying on the fact that water molecules are polar, SEF reorients water molecules according to the direction of the electric field, which results in the reduction of the entropy and free energy of the system [132]. In combination with freezing, applying SEF mitigates the formation of large ice crystals, enhances ice nucleation, and lowers the supercooling temperature of the system [133]. Xanthakis et al. [134] observed a decrease of ~56% in ice crystal size of pork tenderloins subjected to SEF with a magnitude of 12 kV compared to untreated samples. Further, histological analysis of these SEF treated meat samples indicated minimal mechanical damage by ice crystals. In another study that examined the effect of freezing with or without SEF on lamb quality, a reduction in drip loss and no effect on color was observed in the SEF treated samples [135]. However, successful application of this technology is dependent on the magnitude of the SEF being applied, which itself is affected by the inherent properties of the food system being evaluated [132]. Hence, additional research is warranted to obtain more definitive information regarding the applicability of this technology.

The use of ultrasonic technology has been recently explored in the area of freezing foods, and has shown promising results with enhancing the stability of frozen food products [136]. Among the ultrasonic technologies that exist, power ultrasonication has been utilized to improve tenderness and color of fresh meat, with a growing interest in the area of frozen foods. Power ultrasound is a technology that utilizes low frequency and high power to generate acoustic waves in a given system. In a liquid medium, power ultrasound can produce cavitating bubbles that oscillate through the liquid, creating micro-streams along the way, which generates strong eddy currents that can distort materials in their surroundings [137]. Moreover, the cavitating bubbles eventually collapse, and generate tremendous force and pressure at local areas. Applying ultrasound during the freezing of meat has been shown to increase the rate of cooling and limit crystal growth [138]. Astráin-Redín et al. [139] observed an 11% decrease in freezing time of chicken breasts when ultrasonication was applied during freezing, with no negative effect on WHC. Similarly, Zhang et al. [140] observed a decrease in the size of ice crystals and drip and thaw losses of pork loin chops subjected to ultrasound-assisted freezing compared to air-frozen chops. Sun et al. [141] suggested that ultrasound-assisted freezing promotes the formation of protein networks within the meat, thereby reducing the mobility of immobilized and free water during freezing and subsequent water loss during thawing. More recently, ultrasound technology has been carried over to assist in thawing meat samples, rather than freezing. Assisted thawing of meat was shown to reduce the thawing time and myofibrillar structural damage caused by freezing [142,143].

## 7. Conclusions

Freezing of meat has been practiced for several millennia as a method of preservation. The freezing process influences the quality attributes of meat, including tenderness, color, WHC, and flavor. In general, freezing positively affects meat tenderness, but impairs WHC and color stability, and negatively impacts flavor. The extent of each of these effects is dictated by the size and distribution of ice crystals that are formed within the meat during freezing. Mitigating the negative effects of freezing on meat quality is a challenge, as ice crystalline size and distribution are governed by a multitude of factors such as freezing rate and temperature and storage duration. Hence, researchers have been exploring different combinations of freezing temperatures, rates, and storage durations in an attempt to optimize the freezing method for better preserving meat quality. Furthermore, novel technologies, such as high-pressure, static electric, and ultrasound-assisted freezing have been exploited in recent years as potential methods to limit the negative effects of freezing on meat. However, most of these applications have not been commercialized for industry use, as more optimization is warranted.

## Figures and Tables

**Table 1:** Effects of freezing rates (slow vs. fast or never frozen) and thawing on meat quality.

Species	Muscle	Rate of Freezing	Storage Temperature & Duration	Thawing and Aging	Outcome (SF vs. FF or NF)	Ref.
Porcine	*Longissimus* steaks 24 h postmortem	SF: −20 °C blast freezer FF: −80 °C liquid nitrogen	Stored at −20 °C for 6-8 weeks	Thawed at 1 °C for 2 days Aging time: 19 days	↓ WHC ↑ Myofibrillar damage No difference WBSF No difference LO	[47]
Porcine	*Longissimus* steaks 24 h postmortem	SF: −22 °C blast freezer FF: −22 °C immersion freezing	Stored at −18 °C for up to 91 days	Thawed at 4 °C for 12 h No aging	↓ WHC ↑ Myofibrillar damage No difference in L* a* b* ↓ WBSF ↑ LO	[48]
Porcine	*Longissimus* steaks 24 h postmortem	^†^ SF: −20 °C ^†^ FF: −80 °C	Stored at −20 °C for 30 months	Thawed at 5 °C for 16 h No aging	↑ WHC ↓ Myofibrillar damage	[49]
Bovine	*Longissimus* steaks 48 h postmortem	SF: −20 °C blast freezer NF	Stored at −20 °C for 8 weeks	Thawed at 4 °C for 16 h Aging time: Up to 7 days	↓ WHC ↓ WBSF ↓ Juiciness	[50]
Bovine	*Longissimus* muscle 24 h postmortem	SF: −18 °C air freezer NF	Stored at −18°C for up to 9 months	^‡^ Aging time: 2 weeks	↓ WHC ↓ WBSF ↓ L* No difference in a* & b*	[51]
Bovine	*Longissimus* steaks 24 h postmortem	SF: −18 °C air freezer FF: −18 °C immersion tank	Stored at −18 °C for 2 weeks	Thawed at 3 °C Aging time: Up to 4 weeks	↓ WHC No effect on WBSF	[52]
Bovine	*Longissimus* steaks 24 h postmortem	^†^ SF: −20 °C NF	Stored at −20 °C for up to 90 days	Thawed at 4 °C for 48 h Aging time: 3 & 10 days	↓ WHC ↑ Tenderness (trained sensory panelists) ↓ L* a* b*	[53]
Ovine	*Longissimus* muscle 24 h postmortem	SF: −18 °C air freezer FF: −18 °C immersion tank	Stored at −18 °C for 2 weeks	Thawed at 3 °C Aging time: 2 weeks	↓ WHC ↑ WBSF SF & FF ↓ L* a* b*	[54]
Ovine	*Longissimus* steaks 18 h postmortem	SF: −30 °C air freezer FF: −80 °C liquid nitrogen	Stored at −18 °C up to 6 months	Thawed at 3 °C Aging time: 72 h	Consumer panelists did not detect any sensory differences	[55]
Ovine	*Longissimus* steaks 24 h postmortem	SF: −18 °C air freezer NF	Stored at −18 °C for 1 week	Thawed at 3 °C Aging time: Up to 14 days	↓ WHC No difference in WBSF ↓ L* & b* No difference a*	[56]
Broiler	*Pectoralis minor* 24 h postmortem	SF: −30 °C air freezer FF: −70 °C liquid nitrogen	Stored at −30 °C for 12 months	Thawed at 2 °C for 24 h No aging	↓ WHC No differences in L* a* b*	[57]
Broiler	*Pectoralis major* immediately after harvest	^†^ SF: −18 °C ^†^ FF: −40 °C	−18°C & −40 °C for 24 h	Thawed at 4 °C No aging	↑ WHC No difference in WBSF No difference in L* & b* ↑ a*	[58]

SF = slow freezing rate; FF= fast freezing rate; NF = never frozen; ^†^ = no mention of thawing method; ^‡^ = no mention of aging method; ↑ = increased; ↓ decreased; WHC = water holding capacity; LO = lipid oxidation; L* = lightness; a* = redness; b* = yellowness; and WBSF = Warner–Bratzler Shear Force.
